# Therapeutic Targeting of Fibrotic Epithelial-Mesenchymal Transition–An Outstanding Challenge

**DOI:** 10.3389/fphar.2019.00388

**Published:** 2019-04-18

**Authors:** Attila Fintha, Ákos Gasparics, László Rosivall, Attila Sebe

**Affiliations:** ^1^ 2nd Department of Pathology, Semmelweis University, Budapest, Hungary; ^2^ 1st Department of Obstetrics and Gynecology, Semmelweis University, Budapest, Hungary; ^3^ Department of Pathophysiology, International Nephrology Research and Training Center, Semmelweis University, Budapest, Hungary; ^4^ Division of Medical Biotechnology, Paul Ehrlich Institute, Langen, Germany

**Keywords:** epithelial to mesenchymal transition, fibrosis, myofibroblast, repair, chronic injury

## Abstract

Back in 1995, a landmark paper was published, which shaped the fibrosis literature for many years to come. During the characterization of a fibroblast-specific marker (FSP1) in the kidneys, an observation was made, which gave rise to the hypothesis that “fibroblasts in some cases arise from the local conversion of epithelium.” In the following years, epithelial-mesenchymal transition was in the spotlight of fibrosis research, especially in the kidney. However, the hypothesis came under scrutiny following some discouraging findings from lineage tracing experiments and clinical observations. In this review, we provide a timely overview of the current position of the epithelial-mesenchymal transition hypothesis in the context of fibrosis (with a certain focus on renal fibrosis) and highlight some of the potential hurdles and pitfalls preventing therapeutic breakthroughs targeting fibrotic epithelial-mesenchymal transition.

## Myofibroblasts and their Role in Tissue Fibrosis

In recent years, fibrotic disorders gradually earned a well-deserved spotlight as epidemiologic data revealed that nearly 45% of all deaths in the developed world are attributed to chronic fibroproliferative diseases (Wynn, 2007). Just listing fibrotic morbidity is impressive: pulmonary fibrosis, liver cirrhosis, progressive kidney disease, cardiovascular disease, atherosclerosis, and systemic sclerosis of connective tissues of the skin and internal organs. Alone, chronic kidney disease (CKD) accounts for a global and US prevalence of 13% (Thomas et al., 2008; Hill et al., 2016); irrespective of the pathological background and the initial cause, a progressive renal fibrosis is the key finding for CKDs. In addition to specific kidney diseases (chronic glomerulonephritis and polycystic kidney disease), conditions reaching epidemic levels globally such as diabetes or hypertension are the leading causes of CKD. Moreover, the link between wound healing, chronic fibrosis, and cancer progression is also recognized in the literature (Cox and Erler, 2014; Rybinski et al., 2014). Despite the enormous impact on human pathology, there are currently no approved, effective treatment strategies targeting fibrosis. Pirfenidone and nintedanib are the exception with the indication for idiopathic pulmonary fibrosis, however, the clinical experience with these drugs is inconclusive, and the long-term safety and efficacy of these medications is yet to be determined.

During their pathomechanism, most chronic diseases will eventually present excessive tissue scarring as a common feature. There is a well-known physiological process that resembles fibrosis–the wound healing process, which is fundamental for the replacement of damaged tissues following an acute injury. During wound healing following tissue injury, an inflammatory response is switched on where the activated macrophages and neutrophils clean up tissue debris and dead cells. An expansive expression of extracellular matrix components precedes a regeneration phase characterized by the restoration of blood vessels and normal tissue structures in conjunction with the elimination of the granulation tissue. Fibrosis is considered a dysregulated wound healing process, which occurs as a consequence of a chronic injury that persists for several weeks or months, or as a consequence of other chronic diseases. Such an uncontrolled wound healing process leads to the formation and accumulation of a permanent scar tissue, which progressively remodels and later destroys normal tissue architecture. The histological characteristics and regulatory mechanisms of fibrosis are similar across different organs (Wynn, 2007).

It has been shown that the main effector cell type responsible for the physiological accumulation of extracellular matrix in the granulation tissue and for the excess deposition of interstitial extracellular matrix under pathologic conditions is the myofibroblast (Roberts et al., 1997; Powell et al., 1999). Myofibroblasts are specialized fibroblast-like contractile cells exhibiting several ultrastructural features of smooth muscle cells. Myofibroblasts are spindle-shaped cells characterized by the presence of microfilament bundles (stress fibers) and α-smooth muscle actin (SMA). Myofibroblasts have a surface characterized by prominent fibronectin fibrils and fibronexus junctions, and present abundant rough endoplasmic reticulum. SMA-expressing myofibroblasts and fibroblasts are distinct cellular entities, similar to smooth muscle cells, myofibroblasts are characterized by cytoplasmic bundles of contractile microfilaments (Desmouliere et al., 2003). Myofibroblasts are characterized by a higher proliferation rate, migration, cytokine expression, and enhanced interstitial matrix production (Guarino et al., 2009). Myofibroblasts synthesize a series of inflammatory and anti-inflammatory cytokines, chemokines, growth factors, inflammatory mediators, as well as extracellular matrix proteins and proteases. When classifying and characterizing myofibroblasts, vimentin, desmin, and SMA are the three filaments mostly used. There are three indispensable local events required to generate SMA-positive differentiated myofibroblasts: accumulation of the biologically active form of TGF-β1, the presence of specialized ECM proteins like the ED-A splice variant of fibronectin, and high cellular stress rendered by the mechanical properties of the ECM and cell remodeling activity (Eyden, 2001; Tomasek et al., 2002).

Under physiological conditions, large numbers of myofibroblasts accumulate at the sites of ongoing inflammation and repair. Besides matrix production, their role here is to effectively close wounds through their contraction (Hinz, 2010). Under such conditions, myofibroblasts act as repair cells, produce and organize extracellular matrix, and restore tissue integrity after injury. During normal wound healing, myofibroblasts disappear following their apoptosis in parallel to the epithelialization stage of the repair (Gabbiani, 2003). However, in the context of pathological scarring, myofibroblasts create a collagen-rich stiff scar, which disrupts the architecture of tissues and alters the biochemical and biophysical microenvironment, resulting in a dysfunctional tissue. In the long term, deregulated activity of myofibroblasts impairs tissue function and leads to organ failure (Hinz, 2009).

## Epithelial-Mesenchymal Transition as a Source of Myofibroblasts

The cellular origin of myofibroblasts was the focus of basic research for many years. The early view was that fibroblasts were responsible for producing interstitial extracellular matrix under physiological conditions. However, under pathologic conditions, these local fibroblasts proliferated and engaged in excessive fibrogenesis acquiring a highly activated phenotype characteristic of myofibroblasts. This idea was supported by findings indicating the presence of fibroblasts positive for proliferation markers at the periphery of the wound, which acquired additional smooth muscle characteristics during wound healing and progressive organ fibrosis (Grillo, 1963). In the kidneys, the leading role of tubulointerstitial fibrosis (TIF) during CKD was recognized when it was established that there is a strong correlation between tubulointerstitial fibrosis and the decrease of the glomerular filtration rate (Risdon et al., 1968). Here, too, interstitial fibroblasts were considered to be the main effector cells in renal fibrogenesis responsible for excessive matrix deposition. Later, the characterization of a novel fibroblast-specific marker, fibroblast-specific protein 1 (FSP1) led to the birth of a paradigm. When FSP1 expression was assessed, only a limited number of cells stained in normal renal parenchymal tissue. However, in kidneys with ongoing fibrosis caused by persistent inflammation, a high number of FSP1+ fibroblasts were found in the interstitial space, where collagen deposition occurred and in the tubular epithelium adjacent to the inflammation foci. This original observation led to the hypothesis that fibroblasts in some cases arise, as needed, from the local conversion of epithelium (Strutz et al., 1995). This conversion was later identified as an epithelial-mesenchymal transition (EMT). The phenomenon of EMT was already known in the literature as Elizabeth Hay described it as early as in 1968 following observations of cell migration in the primitive streak of chick embryos (Hay, 1968). Originally termed, epithelial-mesenchymal transformation or transdifferentiation, it was renamed as transition to reflect the transient and cyclical nature of this plasticity process during development: epithelial cells can undergo EMT and mesenchymal cells can undergo mesenchymal-epithelial transition (Thiery and Sleeman, 2006). As such, the term transition names a variant of transdifferentiation, and describes the mechanism of dispersing cells in vertebrate embryos during tissue differentiation, during fibroblast formation in injured tissues, or during the early steps of metastases in epithelial cancer (Kalluri and Neilson, 2003).

During EMT, epithelial cells lose the expression of characteristic markers (e.g., E-cadherin and zonula occludens protein-1) and undergo a rearrangement of cell contacts and cytoskeleton. As a consequence, cells lose their epithelial adhesive properties. Later, cells start expressing fibroblast-specific and mesenchymal proteins (e.g., FSP1 and plasminogen activator inhibitor-1), start to synthesize extracellular matrix (e.g., fibronectin), and ultimately differentiate into SMA-positive cells, acquiring the myofibroblast phenotype. In parallel to the disruption of the basal membrane cells acquire enhanced migration and invasion potential. The transition is sequentially orchestrated; cells undergo phenotypic changes according to a defined chronology: the epithelial program is switched off in parallel to the activation of the mesenchymal-fibrogenic program, followed by the activation of the myogenic program. Corresponding to a complete EMT, the process culminates with the appearance of myogenic properties characteristic of myofibroblasts (Yang and Liu, 2001; Gasparics and Sebe, 2018).

EMT was shown to play important roles during embryonic development, cancer progression, and fibrotic disorders of mature organs. EMT has been described in embryonic morphogenesis and organ formation. The role of EMT has been established in lung development and palate fusion (Kaartinen et al., 1995). EMT occurs during the development of endocardial cushions in the atrioventricular canal of the chicken heart (Romano and Runyan, 2000). EMT plays an important role in tumor progression and metastasis formation. During EMT, malignant cells lose their epithelial markers and become motile, EMT being linked to metastasis (Huber et al., 2004). “Fibrogenic” EMT has been shown to contribute to progressive fibrosis of the kidney (Yoshikawa et al., 2007), thyroid gland (Grande et al., 2002), lens (Stump et al., 2006), liver (Sicklick et al., 2006), lung (Kim et al., 2006), and in rheumatic diseases (Zvaifler, 2006).

## Epithelial-Mesenchymal Transition During Renal Fibrosis

In the kidney, tubular epithelial cells play an important role during fibrosis. Proteinuria, high glucose, growth factors, reactive oxygen species, and direct interaction with mononuclear cells are well-characterized stimuli that lead to pro-inflammatory reactions in tubular epithelial cells. The idea that the tubular cells may convert into fibrotic myofibroblasts gained wide acceptance after the original observation was published. In the context of TIF, EMT promotes the progression of the fibrotic disease by generating increased numbers of myofibroblasts, and in parallel, by causing a loss of epithelial cells leading to the destruction of renal tissue architecture. On a functional level, EMT means a loss of function (secretion and absorption), but also a gain of function (fibrogenesis) for the tubular epithelial cell (Quaggin and Kapus, 2011). In a transgenic mouse model of TIF, it was even shown that nearly 40% of fibroblasts may have originated from the tubular epithelium (Iwano et al., 2002). In obstructive nephropathy induced by unilateral ureteral obstruction, Yang and Liu (2001) showed abundant cells co-expressing SMA and tubular markers, indicating a transition state between epithelia and mesenchyme. The clinical relevance of EMT has also been demonstrated in a study characterizing human kidney biopsies: EMT was observed in different renal diseases, independently of histological diagnosis. A strong correlation was found between the number of tubular epithelial cells presenting EMT features and serum creatinine (renal functional impairment). The number of tubular cells with EMT features also associated with the degree of interstitial damage (Rastaldi et al., 2002). Importantly, expression of tubular Snail, a key transcriptional regulator of EMT, was shown to repress the epithelial phenotype and has been observed in areas with significant collagen deposition in patients with renal fibrosis (Boutet et al., 2006). The EMT process during fibrosis is regulated by several cytokines and growth factors (Hay and Zuk, 1995), from which TGF-β1 is the most important regulator. Renal expression of TGF-β1 was shown to be elevated in human diabetic nephropathy (Yamamoto et al., 1993) and TGF-β1 was found to correlate with impaired renal function (Hellmich et al., 2000). Importantly, targeted disruption and inhibition of TGF-β1 signaling protected against renal tubulointerstitial fibrosis and epithelial-mesenchymal transition (Sato et al., 2003; Zeisberg et al., 2003). Even more compelling was the evidence for EMT when it was discovered that the highly similar process originating from endothelial cells, the endothelial-mesenchymal transition (Medici and Kalluri, 2012; Gasparics et al., 2016) contributes to cardiac fibrosis and to a large extent (around 40%) to the myofibroblast pool in renal fibrosis as well (Zeisberg et al., 2007a, 2008).

Following the initial observation that SMA expression occurred in the tubules of the fibrotic kidney, several studies did not find such evidence, questioning the validity of the hypothesis. The initial enthusiasm about the fibrotic EMT hypothesis in the kidney also faced criticism. After a line of confirmatory studies, there were also a series of negative results, where lineage tracing experiments yielded negative results, or simply tubular epithelial cells were found to be negative for EMT marker expression during renal fibrosis. In a model of progressive tubulointerstitial fibrosis in 5/6 nephrectomized rats, tubular epithelial cells were characterized by *de novo* expression of SMA 3 weeks after nephrectomy, in parallel with the disruption of the tubular basement membrane (TBM) (Ng et al., 1998). In human glomerulonephritis, there was a high significant correlation between tubular SMA expression and interstitial fibrosis, interstitial SMA(+) myofibroblast accumulation, deposition of collagen types I and III, tubular TGF-β1 expression, and renal dysfunction (Jinde et al., 2001). Others have found no evidence of tubular SMA expression in the context of renal fibrosis. In an accelerated model of angiotensin II-induced renal fibrosis, the staining of SMA-positive myofibroblasts dramatically increased in the peritubular interstitial spaces 48 h after induction of renal fibrosis with Habu venom plus angiotensin II, but tubular epithelial cells remained SMA-negative (Faulkner et al., 2005). Interestingly, in patients aged 4–44 months suffering congenital nephrotic syndrome of the Finnish type manifested by proteinuria, fibrosis, and inflammation, despite the severe tubulointerstitial fibrosis, tubular epithelial cells did not show transition into myofibroblasts based on vimentin, SMA, collagen, or matrix metalloproteinases 2 and 9 (MMP-2 and -9) expression (Kuusniemi et al., 2005). In a tetracycline-controlled transgenic mouse model, overexpression of transforming growth factor (TGF)-β1 in renal tubules induced widespread peritubular fibrosis. Fibrotic tissue was characterizing the areas between intact tubules. Myofibroblasts were derived from local fibroblasts with no evidence for a transition of tubular cells into myofibroblasts, or for cells transgressing the tubular basement membrane (Koesters et al., 2010). Two lineage tracing experiments also contributed to questioning the validity of the EMT hypothesis. Humphreys and coworkers identified pericytes, but not tubular epithelial cells as the origin of myofibroblasts (Humphreys et al., 2010). In another study, Ksp-cadherin promoter was used to label all renal tubular cells with enhanced yellow fluorescence protein as a permanent marker. After UUO, these cells did not express markers of fibroblasts or myofibroblasts (Li et al., 2010). The conflicting and negative results lead to the conclusion that an EMT is not occurring during kidney fibrosis (Kriz et al., 2011). More recent cell fate tracing experiments evidenced that the myofibroblast pool originated to 50% from local resident fibroblasts, 35% through differentiation from bone marrow, 10% *via* EndMT and 5% *via* EMT (LeBleu et al., 2013).

## The Case for Epithelial-Mesenchymal Transition and Epithelial-Mesenchymal Plasticity

The evidence for EMT is probably not as hazy as suggested by the publications mentioned above. The problems regarding the lineage tracing experiment could have been caused by technical issues widely discussed in the literature (Quaggin and Kapus, 2011). The lack of SMA expression in renal tubular cells during fibrosis could also be explained by a time factor: the fibrotic EMT is presumably a lengthy process in a chronic disease background, which is not entirely corresponding to the accelerated murine disease models, or the cell culture dish modeling, where EMT occurs within 3 days following TGF-β1 treatment (Sebe et al., 2008a). It could well be that by the time ex-tubular cells had undergone EMT, they would start expressing SMA and other definitive markers to the point, where the recognizable renal tissue architecture is long overtaken by a fibrotic mass. There are also some supporting data coming from a different aspect of kidney repair mechanisms.

Along SMA, vimentin expression is also considered to be a marker of EMT. It was shown that vimentin is expressed in injured tubular cells during tubular degeneration in chronic diseases and regeneration in acute tubular injury. Moreover, epithelial and mesenchymal markers could be simultaneously co-expressed (vimentin and keratin) in damaged and regenerating tubular epithelial cells after acute injury (Grone et al., 1987). A similar observation was done with cytokeratin and SMA denoting an intermediate stage of phenotypic change (Jinde et al., 2001), where certain epithelial cells concomitantly express mesenchymal markers as well. This intermediate stage was later defined as a partial EMT, which could bring further meaning to experimental observations in this area: without directly contributing to the myofibroblast population, epithelial cells in this stage could relay signals to the interstitium to promote myofibroblast differentiation in other non-tubular cells, fibrogenesis, and inflammation (Grande et al., 2015; Lovisa et al., 2016). Partial EMT and chronic inflammation together create then a profibrotic milieu that enables collagen-production by various cell types involved in the fibrotic process.

The concept of partial EMT is an elegant solution to some of the problems, which arose in the field. This concept is probably in accordance with certain observations made in the context of cancer evolution. At some time point, it is plausible that epithelial cells express concomitantly epithelial and mesenchymal markers and recapitulate certain aspects of the EMT program, and which state would correspond to the recently termed epithelial-mesenchymal plasticity (EMP) phenomenon (Ye and Weinberg, 2015). The kidney is capable of certain regeneration following acute injury. Genetic fate-mapping techniques were used to show that the regeneration of outer medulla nephrons after ischemia/reperfusion injury is predominantly accomplished by surviving, less-injured tubular epithelial cells (Humphreys et al., 2008). Importantly, the same group evidenced that the outer medulla tubular cell proliferation after ischemic injury occurs by self-duplication of epithelial cells following epithelial dedifferentiation (Humphreys et al., 2011). Following acute injury, tubular epithelial cells recover their functionality following repair. During this dedifferentiation, step tubular epithelial cells undergo a plasticity process acquiring certain mesenchymal characteristics (characterized by vimentin expression) in parallel to a downgrade of the epithelial phenotype, resulting in a “hybrid” cellular phenotype (De Chiara and Crean, 2016). This means that the normal tubular repair and recovery following acute kidney injury undergoes a constant epithelial-mesenchymal-epithelial cycle characterized by epithelial dedifferentiation and re-differentiation that promotes renal healing (Ishibe and Cantley, 2008). Tissue fibrosis originates from a well-balanced protective repair response to tissue injury: fibrosis occurs when the responses to the initial injury become misbalanced and misdirected, and the response to the injury is no longer a self-limiting repair process. Dysregulation of this balanced process caused by repeated, ongoing cycles of tissue damage leads to a progression to chronic kidney disease (Schnaper, 2017). As such, during acute kidney injury, the dedifferentiation and proliferation of surviving proximal tubules is a central mechanism of repair and healing. However, continuous, chronic, and repeated injury induces a persistent dedifferentiation of the proximal tubule, which corresponds to the progressive kidney disease manifested in an epithelial-mesenchymal plasticity state characterized by attenuation of epithelial markers and expression of mesenchymal markers indicative of a partial EMT. As such, tissue fibrosis has a regenerative origin. Thus, the identification of a “point of no return,” which characterizes the switch from a “benign” to progressive tubular dedifferentiation could have wide implications for antifibrotic therapies (Gewin, 2018). Importantly, tubular epithelial cells acquiring a partial EMT program suffer an arrest in the G2 phase of the cell cycle. This cell cycle arrest is TGF-β1, Snai1 and Twist1 dependent, and it limits the repair and regeneration potential of the cells (Lovisa et al., 2015). This growth-arrested cellular state corresponds to a failed re-differentiation in a dysregulated regeneration process and leads to a profibrotic tubular environment (Venkatachalam et al., 2015).

**Figure 1:**
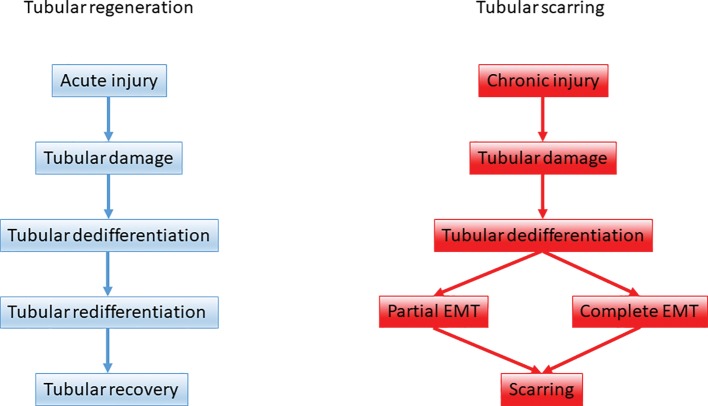
The role of epithelial-mesenchymal plasticity during renal tubular repair and fibrosis. Upon acute kidney injury tubular regeneration takes place through tubular dedifferentiation and redifferentiation on the ground of a balanced epithelial-mesenchymal-epithelial cycle. Upon persistent, repeated injury the dedifferentiation process is hijacked and tubular epithelial cells support scarring via partial and complete EMT.

Throughout the years, evidence mounted for the multitude of cells from which myofibroblasts can originate. It is now clear that local interstitial fibroblasts, vascular pericytes, bone marrow-derived cells (fibrocytes), endothelial and epithelial cells can, to various degrees and through similarly regulated differentiation and dedifferentiation processes, undergo plasticity to give rise to the myofibroblast pool in the kidney (Grande and Lopez-Novoa, 2009). These data in corroboration with other, more recent cell fate tracing evidence give us a complex understanding of the role of EMT during renal fibrosis. There are probably three distinct paths whereby the EMT process is pivotally influencing tissue fibrosis and more specifically kidney fibrosis:

1. There is a balanced epithelial-mesenchymal-epithelial cycle characterizing renal tissue healing during acute kidney injuries. This cycle is marking the tubular dedifferentiation and re-differentiation steps during tubular regeneration.
2. Its dysregulation in the context of chronic renal diseases contributes to fibrogenesis, because EMT contributes to a certain extent to the expansion of the myofibroblast pool.
3. Snail-mediated EMT (partial EMT or epithelial plasticity state) represents a cellular regulatory mechanism, which activates the non-epithelial compartment to promote myofibroblast differentiation and fibrogenesis and to sustain inflammation (Figure 1).

## Role of Fibrotic Epithelial-Mesenchymal Transition in Other Organs

The data on the role of EMT during fibrosis was controversial in other organs as well, and lead to heated debates. Aggregates of proliferating fibroblasts and myofibroblasts characterize pulmonary fibrosis, which is the end stage of tissue responses to injury induced by toxic, autoimmune, and infectious insults (Chapman, 2011). Modeling lung fibrosis in cell fate tracking experiments revealed that vimentin-positive cells in injured lung were mostly of alveolar epithelial origin. This finding was indicative of the epithelial origin of mesenchymal cells during pulmonary fibrosis (Kim et al., 2006). Supportive of the EMT and partial EMT hypothesis, lung epithelial cells from patients suffering idiopathic pulmonary fibrosis were shown to co-express epithelial and mesenchymal markers (Willis et al., 2005; Kim et al., 2006). Later, lineage tracing experiments also confirmed the involvement of EMT in lung fibrosis, although to a lesser extent as in earlier studies (Tanjore et al., 2009). Importantly, significant nuclear β-catenin was detected in bronchiolar and alveolar epithelial cells in biopsies from patients with idiopathic pulmonary fibrosis, suggesting aberrant activation of Wnt/β-catenin signaling. It has been suggested that Wnt/β-catenin signaling would trigger divergent epithelial regeneration and EMT leading to fibrotic remodeling of the lung tissue (Chilosi et al., 2003). Still, the contribution of EMT to lung fibrosis remained controversial. Here as well, a general conclusion is that EMT may provide a direct source of myofibroblasts, however, there is also evidence for the expression of mesenchymal markers in the context of epithelial injury and repair. This aspect, just as in the case of the kidney, makes therapeutic targeting of EMT very challenging and questions the potential therapeutic benefit (Kage and Borok, 2012).

In the liver, the scientific field showed a similar evolution. Liver cells of epithelial origin were suggested to be a relevant source of myofibroblasts. *In vitro* studies demonstrated that treatment of primary rat hepatocytes with TGFβ leads to the downregulation of epithelial markers, the upregulation of mesenchymal marker expression (SMA, collagen, FSP1), and an increase in the migratory potential of cells (Kaimori et al., 2007). Also, lineage tracing experiments evidenced the contribution of hepatocytes to liver myofibroblasts (Zeisberg et al., 2007b). Yet others found no *in vivo* evidence for hepatocytes acquiring a mesenchymal phenotype through EMT during liver fibrosis (Taura et al., 2010). Importantly, just as in the kidney, it was shown, that Snail deletion in hepatocytes attenuates liver fibrosis in adult transgenic mice, an observation also suggesting that hepatocyte-EMT may be involved in liver fibrosis (Rowe et al., 2011).

## Therapeutic Targeting of Epithelial-Mesenchymal Transition and Fibrosis

Throughout the years, several strategies were developed to treat progressive organ fibrosis and EMT. Based on evidence accumulated through clinical and experimental studies, it became obvious that there are several aspects hampering the efficient targeting of this phenomenon.

The problems in the identification of EMT also have ramifications for therapy. First, there is a lack of specific markers to identify and evaluate EMT. The most commonly used markers are mesenchymal of origin and are not exclusively expressed by myofibroblasts: SMA, vimentin, FSP1, fibronectin, collagen I, N-cadherin, Snail, MMP-2, and MMP-9. The concept of epithelial-mesenchymal plasticity or partial EMT further blur the image: cells expressing both sets of epithelial and mesenchymal markers are also difficult to identify in the context of a highly dynamic process that EMT is, and this leads to the problem for therapy as well. Without any specific biomarkers, it is difficult to identify treatment rationales, disease progression, and follow-up. Specific biomarkers with value for treatment schemes need to be identified to precisely describe the different cellular entities and phenotypes arising during a complete or a partial EMT, during the epithelial-mesenchymal plasticity phenomenon. It has been suggested that vimentin and β-catenin tubular staining could be used for screening of early tubular injury in kidney-graft biopsies (Galichon and Hertig, 2011). Maybe not this particular combination (vimentin lacks specificity as a mesenchymal marker), but a combination of both epithelial and mesenchymal markers could shed some light on the complexity of EMT identification. Besides the cellular and mechanistic heterogeneity, patient and disease heterogeneity further complicate the task for the development of antifibrotic, anti-EMT treatments. A more in-depth characterization of the EMT and fibrotic process in close consideration of normal tissue regeneration mechanisms is required to identify novel biomarkers that characterize different key stages, molecular tipping points that could be therapeutically targeted or could be used as accurate clinical endpoints for treatment schemes. The ideal biomarker in this setting would provide useful readouts on clinical efficacy and distinguish between patient strata depending on the response to the therapy.

Besides the biomarker conundrum, there are also other aspects of EMT, which could hamper developing therapeutic strategies. A potential EMT drug must have a broad specificity to cover all mechanisms leading to myofibroblast differentiation from all the different potential cellular sources, and it must have a specificity for the pathologic EMT phenomenon, not interfering with physiological forms of cellular plasticity (exemplified by normal wound healing). These might pose huge obstacles toward specific targeting of fibrotic EMT since the regulation of EMT is highly complex and EMT can be induced by a wide range of stimuli and mediators (Lamouille et al., 2014; Nieto et al., 2016). Among the signaling pathways involved in regulating EMT during renal fibrosis, three turned out to be essential in relaying upstream signals: TGFβ/Smad, Wnt/β-catenin, and integrin/ILK signaling. These pathways are interconnected and regulate further downstream pathways and transcription factors required for EMT (Liu, 2010).

TGF-β1 produced by inflammatory cells, fibroblasts, or epithelial cells is the main inducer of EMT. An important strategy was to target TGF-β and achieve attenuation of EMT and fibrosis by inhibiting TGF-β. There are now several clinical trials reported or still ongoing with the goal to test novel drugs targeting organ fibrosis. One recurring target of these trials is TGF-β inhibition (Li et al., 2017a; Allinovi et al., 2018). Despite intensive research and developments, there are limited achievements coming from this direction. For example, pirfenidone is a drug already marketed with the indication for idiopathic pulmonary fibrosis. The clinical experience is however controversial (Levin and Beaulieu, 2011; Raghu and Thickett, 2013), despite data showing efficient inhibition of experimental fibrosis and EMT in various organs (Macias-Barragan et al., 2010; Li et al., 2017b). Pirfenidone has been assessed in diabetic nephropathy patients as well. The high rate of dropouts are concerning; it is also puzzling to see the lack of effect of the suggested maintenance dose (Sharma et al., 2011). Currently, there is only one therapy used as the standard of care in the case of renal fibrosis: ACE inhibitors have demonstrated protective renal effects and can ameliorate fibrosis (Brenner et al., 2001; Lewis et al., 2001). However, this treatment cannot completely suppress the progression of renal disease. Despite the fact that this effect is partly manifested by an inhibition of angiotensin II mediated expression of TGF-β1 (Kyuden et al., 2005; Peng et al., 2005), a recent clinical trial combining ACE inhibitors and TGF-β1-specific, humanized neutralizing monoclonal antibody had to be stopped early due to futility. This combination did not slow the progression of diabetic nephropathy and it seems that inhibition of TGF-β1 alone is not sufficient to induce further improvements in patient outcomes (Voelker et al., 2017).

Targeting TGF-β may also interfere with physiological mechanisms, since TGF-β is also involved in normal wound healing, immune regulation, and tumor suppression (Gabbiani, 2003; Bierie and Moses, 2006; Li et al., 2006). Influencing TGF-β expression or systemic inhibition of TGF-β may induce potential adverse effects. A more selective approach could be required to exploit the central role of TGF-β1 in the induction of EMT and fibrosis. One aspect of fibrosis is a systemic, but also a local overexpression of latent, inactive TGF-β. TGF-β can be locally activated, for example, by tissue stiffness (Hinz, 2009). This could mean a point of intervention, whereby reducing tissue stiffness could lead to less active TGF-β. Such an approach would complement for strategies adjusting the level of TGF-β1 to a physiological one.

Another desirable alternative would be to target the principal downstream pro-fibrotic and EMT-inducing effectors of TGF-β. The feasibility of such interventions has been shown: inactivating Snail1 in mice with established renal fibrosis improves organ morphology and function, significantly attenuating the disease (Grande et al., 2015). Nevertheless, caution has to be applied for such applications as well. In our earlier work, we identified the pivotal role of the myocardin-related transcription factors (MRTFs) during EMT, and this was later confirmed by other groups as well (Gasparics and Sebe, 2018). TGF-β1 and small GTPases regulate the function of MRTF during EMT of tubular epithelial cells (Fan et al., 2007; Sebe et al., 2008b, 2010). MRTFs are also influencing the expression of all major EMT markers and transcriptional regulators, and all three major EMT pathways delineated for renal fibrosis: TGFβ/Smad, Wnt/β-catenin, and integrin/ILK signaling (Gasparics and Sebe, 2018). There is even an endogenous mechanism inhibiting MRTF: by interfering with MRTF function SCAI inhibits EMT induced by TGF-β1 and angiotensin II (Fintha et al., 2013; Gasparics et al., 2018). Inhibition of MRTF was even shown to prevent lung fibrosis in two distinct murine models of fibrosis (Sisson et al., 2015). Yet these data are still not sufficient to indicate MRTF as a target for therapy in the context of organ fibrosis and EMT.

MRTFs are also important regulators of normal physiological processes. MRTF-B plays an essential role during smooth muscle differentiation (Oh et al., 2005), and RhoA-dependent smooth muscle cell-specific transcription is mediated by increased nuclear translocation of the MRTFs (Hinson et al., 2007). MRTF-A regulates capillary proliferation and pericyte recruitment while promoting neovascularization in ischemic hindlimb models (Hinkel et al., 2014). MRTF-A is a key regulator of mammary gland functions and is essential for the maintenance of lactation (Sun et al., 2006). MRTF-A is abundantly expressed in the newborn rat cortical and hippocampus neurons and in adult rat forebrain neurons. Its overexpression in the brain inhibited neuronal apoptosis (Cao et al., 2011). MRTF-dependent signaling is activated in response to skin injury, being an essential step mediating wound closure, wound healing and suppression of the inflammatory response (Velasquez et al., 2013). MRTFs are crucial in the hematopoietic system as well. MRTFs regulate hematopoietic stem cell progenitor cell homing into the bone marrow (Costello et al., 2015) and play a crucial role in megakaryocyte maturation and platelet formation (Smith et al., 2012). MRTF-A regulates the normal function of lymphoid and myeloid lineage immune cells (Record et al., 2015). Therefore, the specificity requirement could not be fulfilled by systemic targeting of MRTFs or any other similar targets with wide implications for normal cellular functions. The requirement of exclusivity and specificity for targeting EMT and fibrosis are also mirrored by several clinical trials discontinued and prematurely stopped due to adverse events (Allinovi et al., 2018).

One strategy to relieve organ fibrosis could be the induction of apoptosis in the myofibroblasts. Such an approach would partly mimic physiological events occurring during wound healing, where myofibroblasts disappear by apoptosis when epithelialization occurs (Gabbiani, 2003). There are different approaches to induce myofibroblast apoptosis. It has been shown that bFGF can promote scarless wound healing upon the induction of apoptosis of myofibroblasts. This effect even seems to be specific for myofibroblast, but not fibroblasts (Abe et al., 2012). Also, pharmacologic disruption of mechanosensitive signaling with the ROCK inhibitor fasudil-induced myofibroblast apoptosis. Treatment with fasudil during the post-inflammatory fibrotic phase of lung injury protected mice from experimental lung fibrosis in an MRTF-dependent manner (Zhou et al., 2013). Nonetheless, irrespective of how successful such an approach will be in the future, the repair of the destroyed parenchymal tissue will not take place. This raises the question of whether fibrosis is reversible. Of course, due to its high regenerative potential, liver is not the best example here. Nevertheless, it was shown that by treating viral hepatitis organ function can be restored by resolution of hepatic fibrosis. Arresting the underlying injury, the viral infection, leads to a regression of cirrhosis often characterized by a return to normal liver structure and function (Chang et al., 2010; Ellis and Mann, 2012). It is the reversible nature of the epithelial-mesenchymal-epithelial transitions that makes such reversal of fibrosis possible. There were a number of *in vitro* studies showing that a reversal to the epithelial phenotype is possible in the context of fibrosis: the concept gained more acceptance once it was shown that the systemic administration of recombinant human BMP-7 in a mouse model of chronic renal injury lead to the repair of damaged renal tubular epithelial cells in conjunction with the reversal of the chronic renal injury (Zeisberg et al., 2003). The concept of reversal from the myofibroblast-mesenchymal state to an epithelial phenotype could also be potentially achieved by *in vivo* reprogramming, as recent evidence suggests. Transduction of four transcription factors that specify the skin-cell lineage enabled efficient and rapid *de novo* epithelialization from the surface of cutaneous ulcers in mice *via* the reprogramming and epithelial conversion of wound-resident mesenchymal cells (Kurita et al., 2018). Besides the “classical” methods developed to tackle EMT and tissue fibrosis with chemical compounds or monoclonal antibodies, the antifibrotic therapeutic toolbox may soon include some revolutionary methods worth further investigation. Recently a CRISPR/Cas9-based gene-specific demethylating technology was used to reactivate genes hypermethylated in kidney fibrosis. The reactivation of certain genes in UUO-challenged kidneys led to a marked reduction of fibrosis (Xu et al., 2018). The specificity of the targeted genes in this particular study may be a matter of debate; nonetheless, the technology is very much appealing and could even allow organ specificity of application routes.

The conclusion of this review is on a dim note. We are farther away than ever from the therapeutic targeting of EMT during fibrosis. The whole EMT hypothesis and its role in fibrosis has been reconsidered in recent years, and its parallels to normal dedifferentiation-based healing mechanisms raise specificity concerns: an interference with overlapping mechanisms could lead to side effects manifested in a hampered physiological tissue healing processes in the same or other organs. Our understanding of acute injury repair has made significant process, yet we do not understand how this process gets derailed and turns detrimental in the context of progressive scarring. Also, a multitude of issues are hampering the identification of specific signaling mechanisms and biomarkers which could be exploited for therapeutic purposes. A disentanglement of the different cellular plasticity events and substages in the process of partial EMT and EMP could provide further leads into identifying points of no return or tipping points qualifying as vulnerability spots delineating normal dedifferentiation processed from responses to chronic injury. All in all, more research needs to be carried out to further decipher and identify potential intervention targets, which would overcome many obstacles presented here.

## Author Contributions

AF, ÁG, and AS drafted the manuscript. LR and AS edited and revised the manuscript. AS contributed to the conception and final approval of the manuscript.

### Conflict of Interest Statement

The authors declare that the research was conducted in the absence of any commercial or financial relationships that could be construed as a potential conflict of interest.
